# Evaluation of pain intensity and airway changes in non-growing patients treated by MARPE with and without micro-osteoperforation: a randomized clinical trial

**DOI:** 10.1186/s12903-024-05196-4

**Published:** 2024-11-20

**Authors:** Moataz Elshehaby, Nehal Fouad Albelasy, Mohamed A. Elbialy, Ahmad Mohammed Hafez, Yasser Lotfy Abdelnaby

**Affiliations:** 1 Department of Orthodontics, Faculty of Oral and Dental Medicine Dentistry, Delta University for Science and Technology, Algomhoria St, Mansaura, 35116 Egypt; 2 Department of Orthodontics, Faculty of Dentistry, Mansoura University, Mansoura, Egypt

**Keywords:** MARPE, Micro-osteoperforation, Maxillary transverse deficiency, Pain, Airway volume, Randomized clinical trial

## Abstract

**Trial design:**

Parallel.

**Objectives:**

To assess the effect of mini-screw assisted rapid palatal expansion (MARPE) with/without micro-osteoperforation (MOP) on the airway and pain intensity in non-growing patients with maxillary transverse deficiency.

**Method:**

Two equal groups of twenty-four individuals aged ≥ 19 years old with maxillary transverse deficit were randomly assigned. MOP-facilitated MARPE was used to treat one group (MMG), and the other group was treated with MARPE without MOP (NMG). For airway evaluation, CBCT images were obtained 2 months before starting the palatal expansion and 3 months after finishing the expansion in 28 days. The Visual Analogue Scale (VAS) was used to measure the pain level.

**Results:**

Significant suture opening was observed in both groups. All linear measurements of the nasal cavity and volumetric measurements of the nasal passage and oropharyngeal airway increased significantly in both groups, with no significant difference between them. Moderate pain was experienced in the first two weeks of expansion in MMG (5.11 ± 0.30), while more significant pain was recorded in NMG (6.87 ± 0.40). Pain decreased significantly in the following two weeks in MMG (2.77 ± 0.39) and in NMG (5.11 ± 0.32), with a significant difference between the two groups throughout the entire duration of expansion.

**Conclusion:**

Transverse maxillary deficit was successfully treated with both expansion methods, with and without MOP, with comparable skeletal effects at the nasal levels and airway volumetric improvement. So, MOP did not provide any further advantage in improving the airway volume after maxillary expansion. However, it significantly reduced pain intensity throughout the entire duration of expansion.

**Trial registration:**

The protocol registration and results system (PRS) of ClinicalTrials.gov has this RCT registered under the number NCT06502041 on 13/07/2024.

## Background

One of the goals of rapid palatal expansion (RPE), an orthodontic method that is frequently employed to repair transverse maxillary deficit, is to enhance breathing through the nose [1, 2]. For children and adolescents whose intermaxillary suture has not been fully osseous, conventional RPE is recommended. With adults, however, this kind of therapy is typically not used for expansion due to the more rigid midpalatal suture (MPS) interdigitations [1]. Consequently, rather than causing actual bone expansion in adults, the expansion forces applied to teeth in conventional RPE devices might cause unfavorable dental consequences such as dental tilting and alveolar bone bending [3]. Moreover, the limitations and adverse consequences of RPE are frequent and include restricted or failed skeletal expansion, discomfort, tissue oedema, inclination of the posterior teeth buccally, root resorption, gingival recession, and relapse [4].

Although the MARPE approach may represent a non-surgical alternative to treating maxillary deficit in young people during the late stages of growth and adults, it may exhibit difficulties owing to the increased interdigitation of the MPS that happens following puberty [5]. Force should be sufficient to overcome midface resistance regions, including zygomatic buttresses, pterygoid junctions, piriform aperture pillars, and MPS which needs to be broken in order to achieve MARPE skeletal outcomes [6].

A few case studies [7, 8] in the literature showed how the corticotomy technique—also known as corticotomy-assisted expansion, or CAE—was used to help expand the upper arch in patients with maxillary transverse insufficiency. In addition to using dental expanders, the approved corticotomy procedure entails bilateral decortication of the palatine, buccal, and alveolar bones. According to Hassan et al. [9], the corticotomy technique during expansion can lower the adverse consequences of traditional expansion, increase tooth movement more quickly, and decrease resistance to expansion. An adult patient treated with corticopuncture (CP) aided MARPE was described in a recent case report [10]. Micro-osteoperforation (MOP)/CP was performed along MPS using a 1.1mm diameter and 4mm bur to reduce the resistance and optimize its opening.

The effect of MARPE on the upper airway was demonstrated in recent researches [11–13] and it was found that MARPE considerably enhances in younger patients, the minimum cross-sectional area and the airway capacity. However, no previous study evaluated the effect of MOP-facilitated MARPE on the airway.

For better patient experience, education, and pain management, clinicians should be aware of the pain degree and discomfort that patients feel. This should be thoroughly assessed. However, a recent review of the literature [14] found that there is a dearth of research on the subject of the sensation of pain during maxillary expansion using MARPE.

Thus, the purpose of this study was to assess the effect of MOP-facilitated MARPE on the airway compared to MARPE without MOP on non-growing patients using cone-beam computed tomography (CBCT) and to evaluate pain intensity in both situations.

## Materials and methods

### Trial design

This is a prospective, single-centre, two-arm, parallel-randomized clinical trial with a 1:1 allocation. The assignment of patients to the comparison and intervention groups was random and made as follows:

a MARPE with MOP group: MMG.
b MARPE with no MOP group: NMG.

This randomized clinical study was registered on July 13, 2024, via the protocol registration and results system (PRS) of ClinicalTrials.gov under the number NCT06502041.

### Ethics approval and consent to participate

This study was approved by the "Dental Research Ethics Committee" of the Mansoura University Faculty of Dentistry. Patients were recruited from the orthodontic department's outpatient clinic at Mansoura University's Faculty of Dentistry between September 2022 and January 2024. Each recruited patient signed the informed consent form after learning about the intervention's purpose and associated risks and benefits.

#### Inclusion criteria

1. Post-pubertal adolescent aged 20–25 years old.
2. Skeletal transverse deficiency with unilateral or bilateral crossbite with maxillary mandibular width difference per side not more than 5mm.
3. The emergence of the upper permanent dentition up to the second molars
4. Crowding in the upper arch is less than 8mm

#### Exclusion criteria

Patients with conditions or medications that affect the bone, previous orthodontic treatment, active periodontal disease, oral habits, craniofacial congenital anomalies, such as cleft lip and palate, and pregnant females were excluded.

### Intervention

**Fig. 1:**
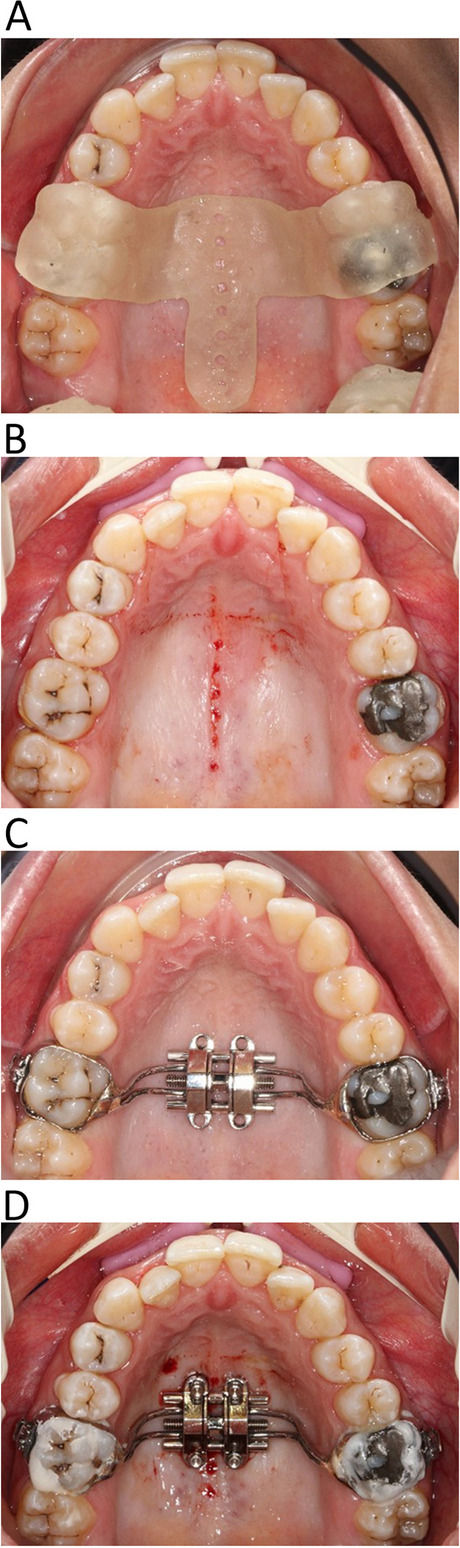
**A** surgical guide in situ. **B** 8 bone perforations 2mm apart 5 mm deep, were performed along the MPS with the aid of a surgical guide, prior to the insertion of the MSE. **C** MSE insertion and cementation. **D** Four mini-implants of 1.8 mm diameter and 11 mm length for the anterior region and 13 mm for the posterior one were inserted into the tubes and passed through palatal cortical bone under local infiltration anesthesia

The first stage of treatment for each patient in the two groups involved maxillary arch extension using the 10 mm Maxillary Skeletal Expander® "MSE II" (Biomaterials Korea, Seoul, South Korea). To ensure that it was precisely tailored for each participant in the laboratory, Brunetto et al. [15] recommendations were followed: orthodontic bands were fitted on permanent first upper molars; alginate imprints were taken; plaster was poured, and the bands were transferred; the wire was bent, and the bands were soldered on plaster castings; expander was assembled intraoral. The appliance was cemented to the first molars using glass ionomer luting cement (GC Fuji I, Tokyo, Japan). Four mini-implants (Biomaterials Korea, Seoul, South Korea) of 1.8 mm diameter and 11mm length for the anterior region and 13mm for the posterior one were placed into the tubes passing through palatal cortical bone after local infiltration anesthesia, as shown in Fig. 1.

The activation protocol was two-quarter turns (0.26 mm, 0.13 mm per turn) every 12 h (4 times per day) for the first two weeks. Then, for the following two weeks, it was activated one-quarter turn every 12 h (2 times per day) till full expansion in 28 days.

#### For the MMG

Using ring infiltration anesthesia, eight bone perforations 2mm apart, 5 mm deep [16], were performed along the MPS with the aid of a surgical guide from CBCT prior to the insertion of the MSE, as shown in Fig. 1a&b. The procedure was carried out manually through the insertion and removal of a 9mm titanium alloy mini-screw with a 5mm double thread, 4mm neck length, and 1.8mm diameter.

#### Adherence

1. Patients were requested to attend follow-up appointments on a regular basis, and their attendance at these appointments was regularly tracked.

2. Patients should strictly avoid any drug intake during the study except after the check that it does not affect the treatment protocol.

#### For the NMG

 The same procedures as in the MMG were followed but without micro-osteoperforations.

For each patient, full skull CBCT was obtained two months before starting the palatal expansion and three months after finishing the expansion in 28 days from its beginning with the expander in situ, by means of an iCAT next generation (Imaging Science International (ISI), Hatfield, PA, USA). The scan settings were 120 kV, 5 mA, 8.9 s of exposure time, 13 cm of height and 16 cm of diameter, and 0.3 mm of voxel size. The head and tongue were in a standardized position during the image acquisition and the patients were instructed to swallow before the image acquisition and to avoid swallowing during the image acquisition. The Digital Imaging and Communications in Medicine (DICOM) file format was used to reconstitute the CBCT data. The same researcher performed the volumetric assessment of the airways in the pre- and post-MARPE photos, utilizing Invivo software (Anatomag version 6, Santa Clara, CA, USA) and Ondemand 3D software V1.0.10.7510 (Cybermed – Seoul, Korea).

### Outcomes

#### Airway

Airway linear and volumetric measurements are illustrated in Table 1.

**Table 1:** Airway linear and volumetric measurements

AOS (anterior opening of the suture)	Opening distance of the midpalatal suture at the level of the canines	
POS (posterior opening of the suture)	Opening distance of the midpalatal suture at the level of the palatal roots of the first molars	
Nasal cavity width (NCW)	Linear distance between the left and right points at the lowest part of the maximum concavity of the piriform rim (Fig. 2)	
WAR (Width of the anterior region of the nasal cavity)	Distance between the most antero-inferior points of the piriform aperture on each side, in the axial plane (Fig. 3)	
WMR (Width of the middle region of the nasal cavity)	Distance between the lateral walls of the nasal fossa on each side, 15 mm posterior to WAR (Fig. 3)	
WPR (Width of the posterior region of the nasal cavity)	Distance between the lateral walls of the nasal fossa on each side, 15 mm posterior to WMR (Fig. 3)	
**Volumetric measurements**		
	Nasal passage volume (NPV) (Fig. 4)	Oropharyngeal airway volume (OPV) (Fig. 5)
Anterior limit	Soft tissue of the nose	Straight line that passes through PNS, at 90° with the lower limit of the OP
Posterior limit	Posterior wall of the nasopharynx soft tissue	Posterior wall of the oropharynx soft tissue
Superior limit	Point where the nasal septum merges with the upper wall of the nasopharynx	Line that divides the pharyngeal airspace from the palatine plane (ANS–PNS)
Inferior limit	Line that divides the pharyngeal airspace from the palatine plane (ANS–PNS)	Line parallel to the palatine plane (ANS–PNS) that passes through the superior border of epiglottis
Lateral limit	Manually delimited to isolate the maxillary sinus ostia	Lateral walls of the oropharynx soft tissue
**Min area** Minimum constricted area	Minimum constricted area of airway path (Fig. 5)

**Fig. 2:**
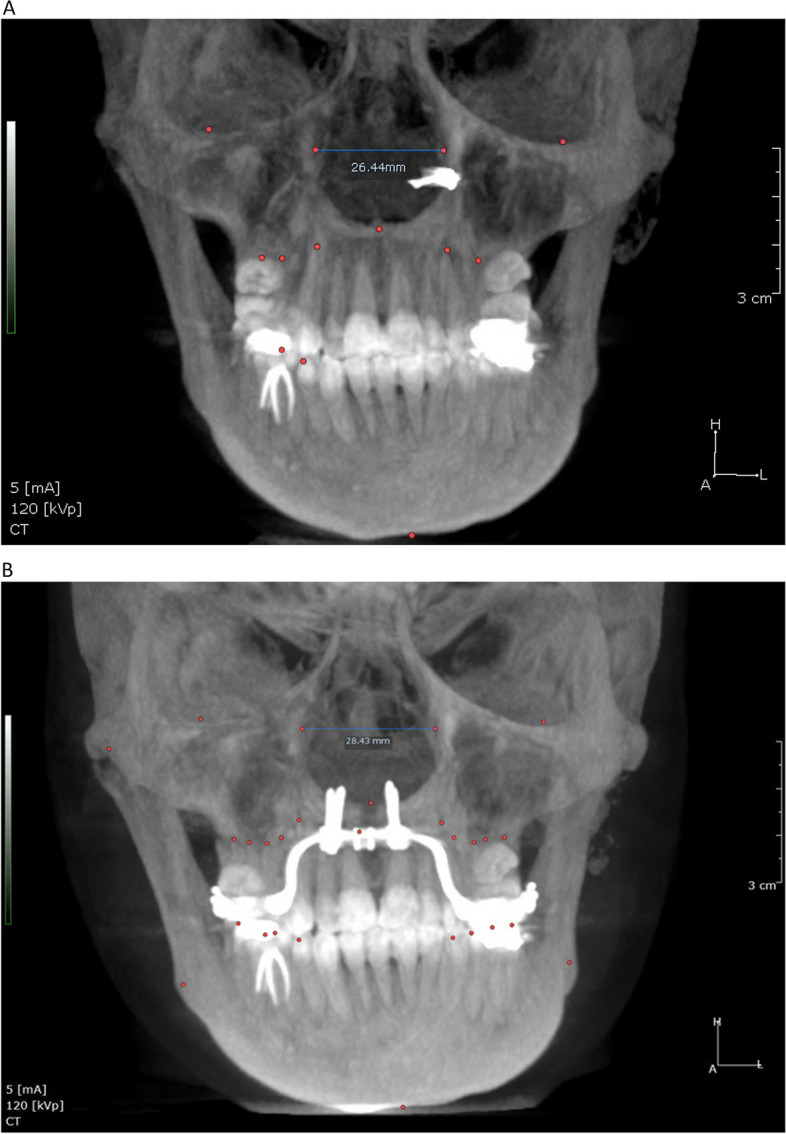
Nasal cavity width (NCW). **A** before expansion, **B** after expansion

**Fig. 3:**
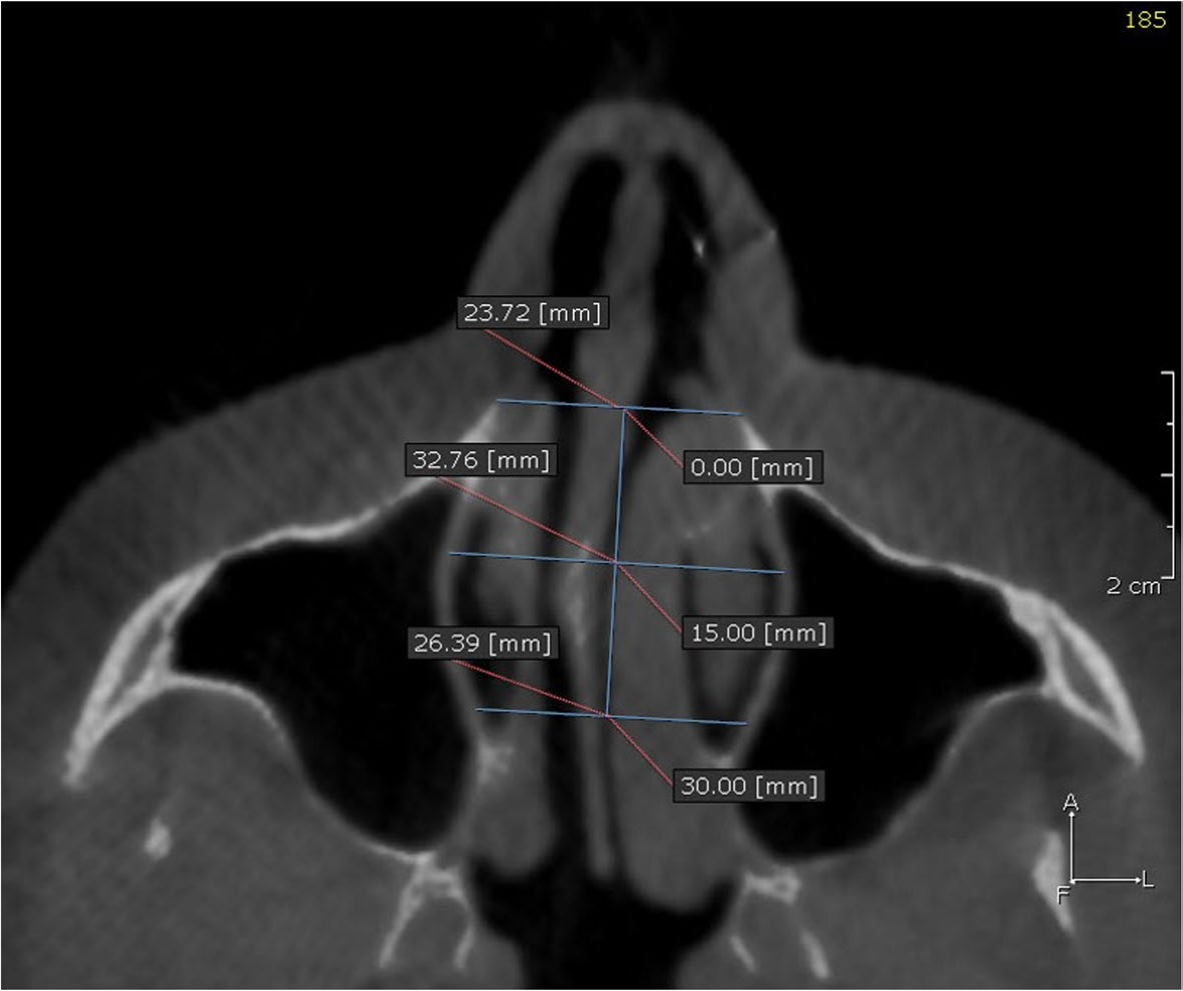
WAR (Width of the anterior region of the nasal cavity), WMR (Width of the middle region of the nasal cavity), and WPR (Width of the posterior region of the nasal cavity)

**Fig. 4:**
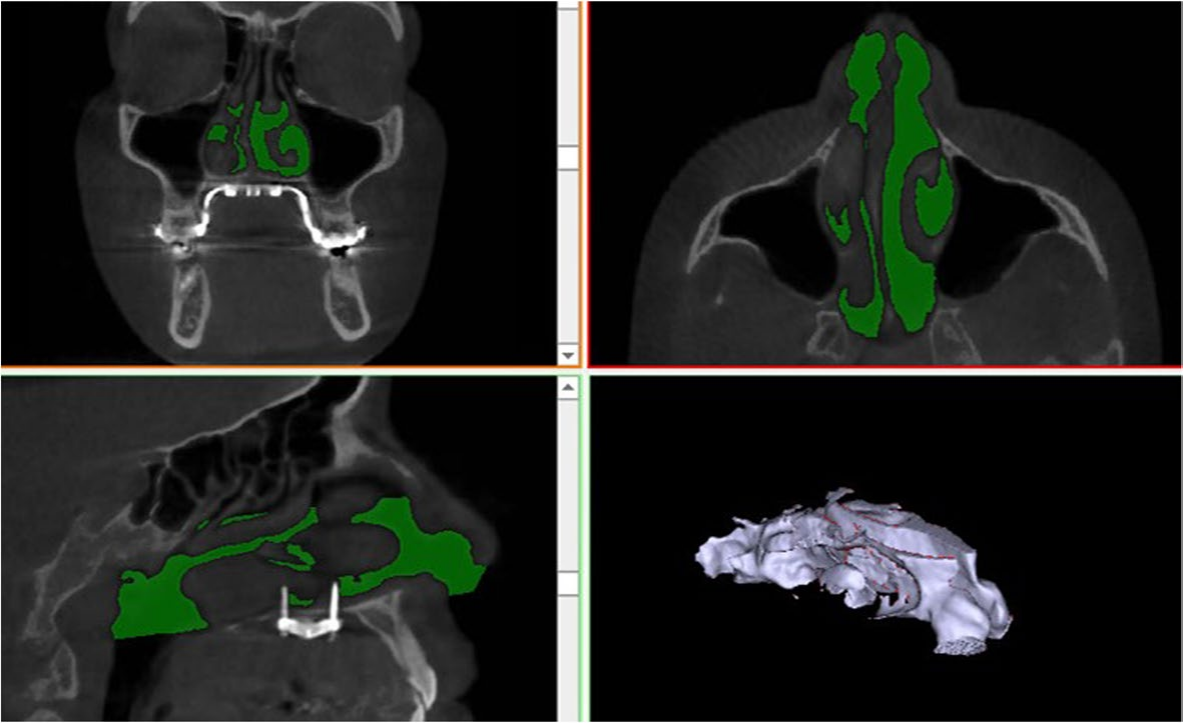
Nasal passage volume (NPV)

**Fig. 5:**
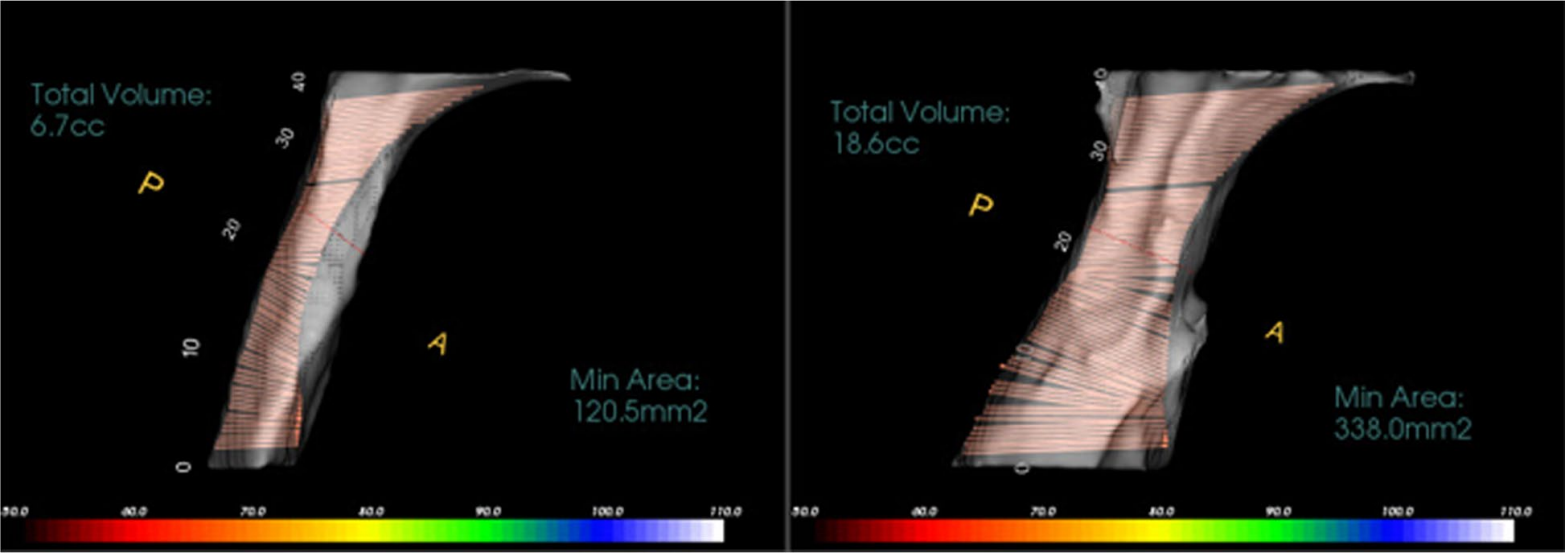
Oropharyngeal airway volume (OPV) and minimal constricted area before (left) and after (right) expansion

#### Pain

**Fig. 6:**
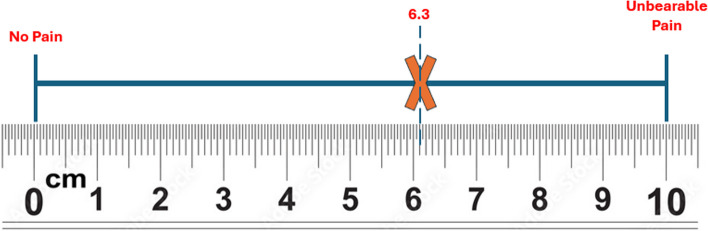
The Visual Analogue Scale (VAS)

Pain was assessed using the Visual Analogue Scale (VAS), a 100-mm line with "no pain" at the left end and "the worst unbearable pain" at the right, which corresponds to a pain score between 0 and 100mm. Patients marked their level of pain using this single-dimensional pain scale. When the cause of acute pain is known, this validated technique can be used to measure the severity of pain as well as assess any variations and clinically significant intraindividual changes in pain intensity [17]. All participants were asked to place a vertical mark indicating the level of pain on the VAS 4 h after each activation (two times per day) for 28 consecutive days of activation using a pain diary sheet containing the actual size of a 100mm of VAS. Then the mark was compared to a calibrated 10 cm ruler to define the number in mm on the scale as shown in Fig. 6. If a participant experienced unbearable pain, the participant could take a rescue dose of analgesic and was considered a dropout participant after that.

### Sample size calculation

G*Power 3.1.9.4 (Kiel University, Germany) was used to estimate the sample size. Based on the earlier research by de Oliveira et al. [18] this study was planned to have a power of 90%, assuming a type I statistical error of 5% and a two-tailed statistical test. The mean changes in posterior maxillary base were 2.27 ± 1.10 mm and 0.11 ± 0.63 mm for the MARPE and SARPE groups, respectively. The calculated sample size was six patients per group. To guard against the dropouts, the sample size was increased to 12 patients per group. Since we had a trial design with a 1:1 allocation ratio, 24 patients were required for the study.

### Randomization

Participants were randomly assigned to two intervention groups on a one-to-one basis utilizing the web program RANDOM.ORG for sequence generating.

The concealment was done using opaque sealed envelopes which contained the numbers from 1- 24. Every patient was permitted to select an envelope to find their own individual number. According to the number, patients were assigned to one of the 2 intervention groups.

### Blinding

Blinding of either the patient or clinician was impossible. However, blinding during analyzing the data was ensured. All radiographs were coded and shuffled before they were evaluated and measured.

### Statistical analysis

Utilizing GraphPad Prism 8 (GraphPad Software), data was entered into the computer and analyzed. Mean and standard deviation (SD) values were used to present the data.

To compare the two groups under study, the Unpaired Student T-test was employed, and to compare the two time points within the same group, the Paired Student T-test was utilized. A significance level of 0.05 was used to assess the results.

## Results

### Participant flow

**Fig. 7:**
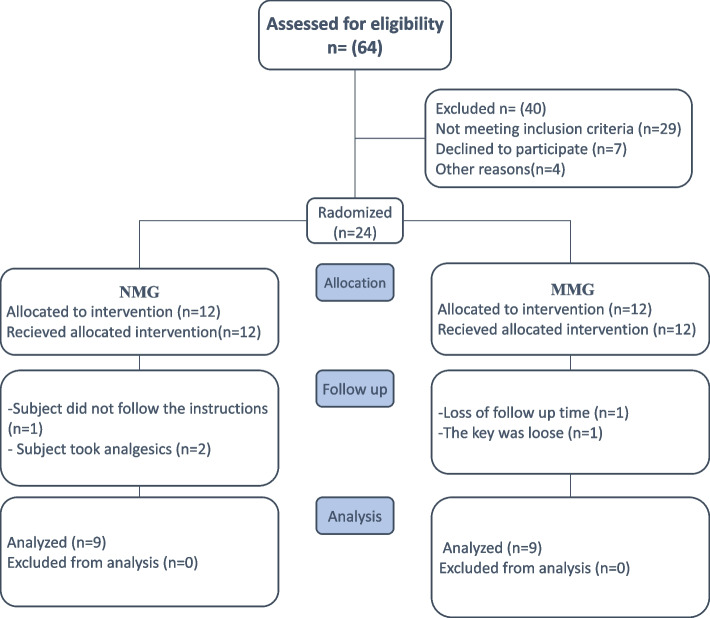
The Consolidated Standards of Reporting Trials (CONSORT) participant flow diagram

**Fig. 8:**
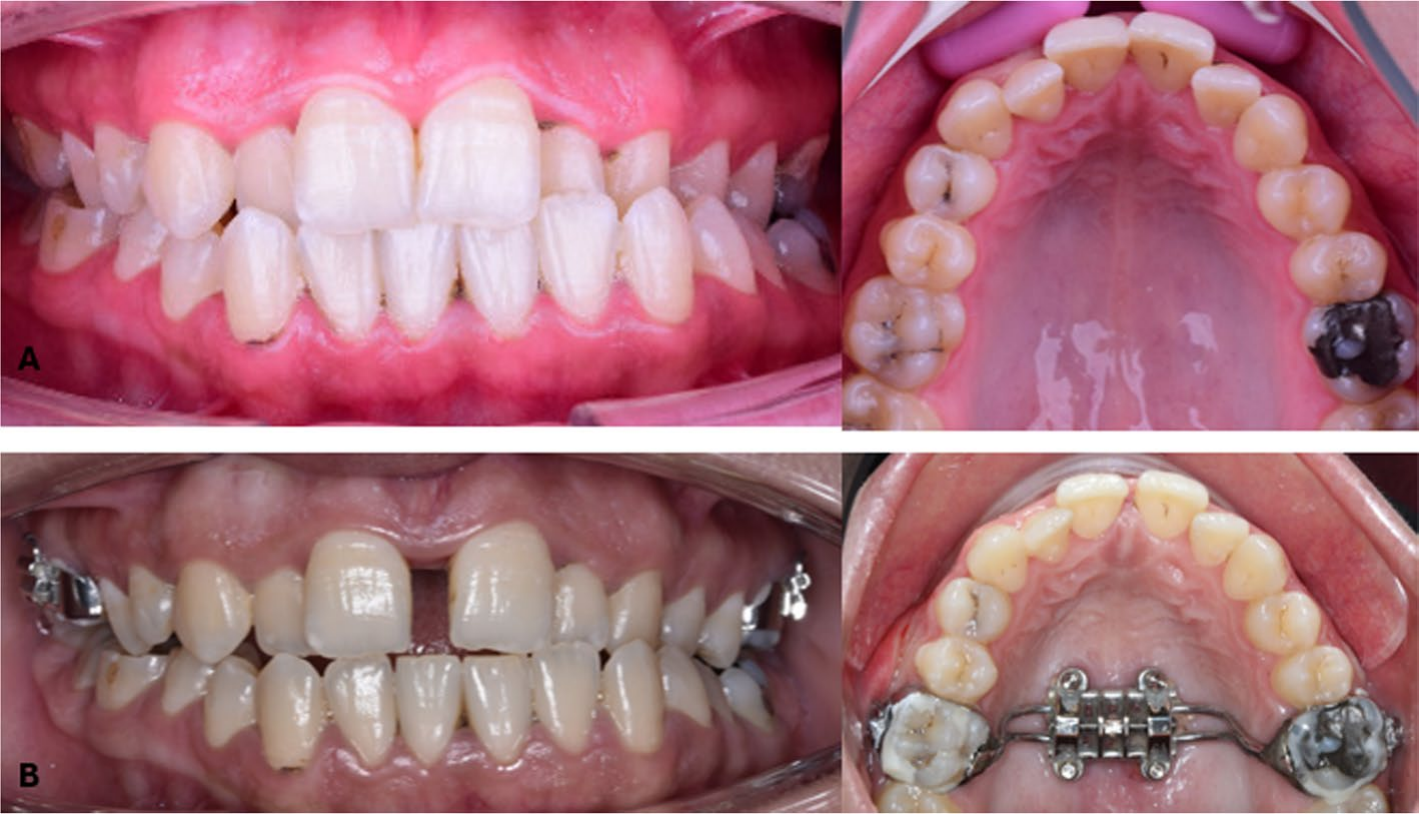
Intraoral photos of a case in MMG; **A** before expansion, **B** after expansion

**Fig. 9:**
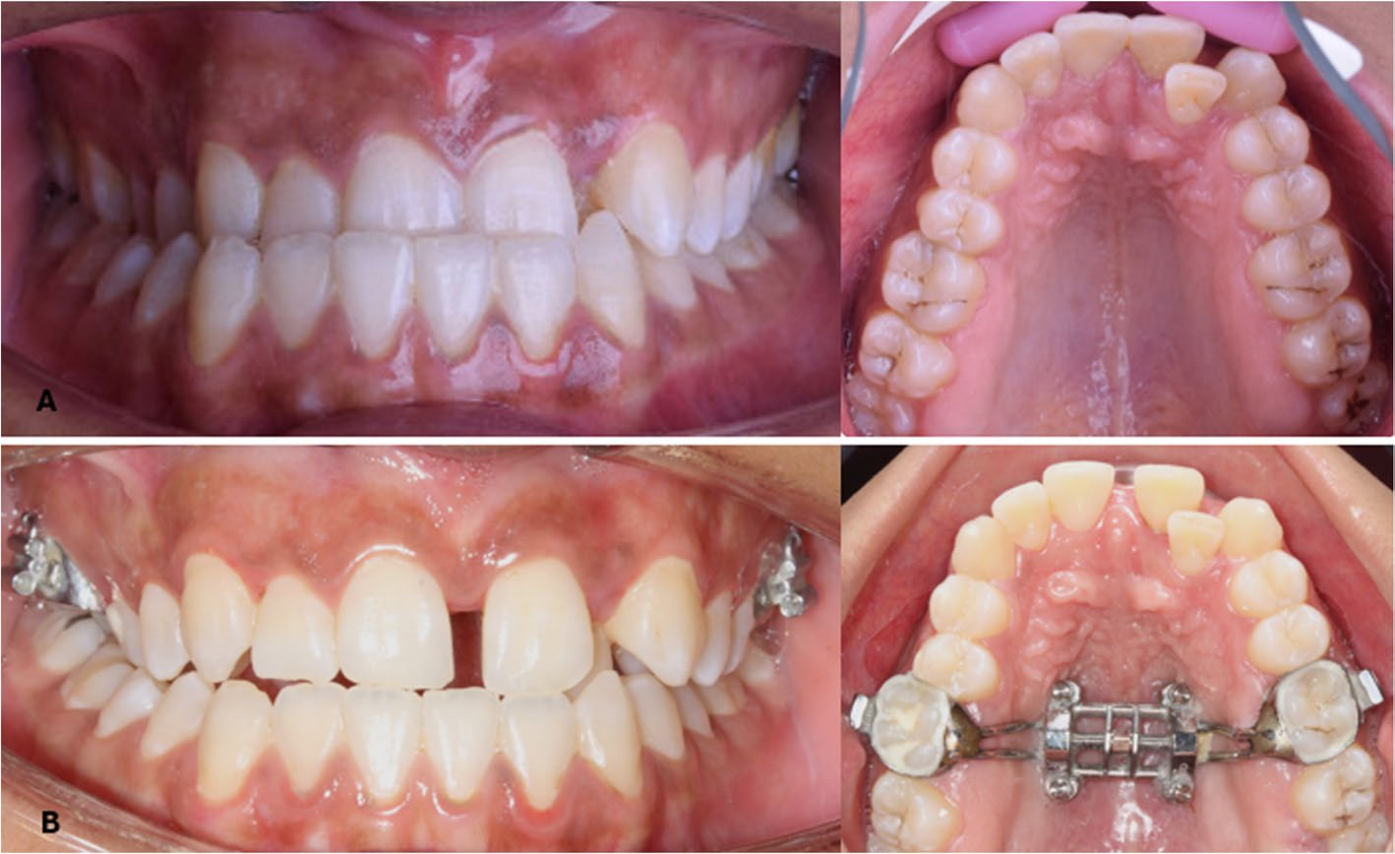
Intraoral photos of a case in NMG; **A** before expansion, **B** after expansion

Recruitment began in September 2022 and continued until January 2023. Twenty-four individuals were enlisted and split into two groups, MMG (n = 12) and NMG (n = 12), in a 1:1 ratio. Three patients dropped out from each group and only 9 patients were analyzed in each group, as shown in Fig. 7. Pre- and post-expansion intraoral photos in both groups are shown in Figs. 8 and 9.

### Baseline data

All the measurements before starting the expansion did not differ significantly between the two groups. There was a statistically insignificant difference between the two groups regarding male and female distribution within each group (4 males and 5 females in each group). The mean age of patients was 20.89 ± 1.17 years in NMG and 21.11 ± 1.45 years in MMG with no significant difference between the two groups.

### Outcomes measurements

#### Airway measurements

With significant suture opening in both groups, all linear measurements of the nasal cavity and volumetric measurements of the nasal passage and the oropharyngeal airway increased significantly in both groups with no significant difference between them. However, the minimum constricted area increased significantly by 149.92 ± 114.69 and 56.21 ± 63.22 cc in NMG and MMG respectively with a more significant increase in NMG. The oropharyngeal airway volume increased significantly by 5.74 ± 3.82 and 5.01 ± 4.91 cc in NMG and MMG respectively. Table 2 displays a comparison of the changes in MMG vs. NMG.

**Table 2:** Comparisons of pre-post change in NMG vs. MMG

Measurement	NMG	MMG	*P* value	95% CI	
AOS (mm)	0.96 ± 0.72	1.46 ± 0.66	.144	−1.19211	.18989
POS (mm)	1.32 ± 0.44	1.54 ± 0.77	.464	-.85985	.41540
NCW (mm)	2.78 ± 1.59	2.35 ± 1.47	.565	−1.10623	1.95512
WAR (mm)	2.57 ± 0.95	2.43 ± 1.4	.804	−1.06564	1.35009
WMR (mm)	2.55 ± 0.91	2.48 ± 1.38	.615	−1.01594	1.65149
WPR (mm)	2.68 ± 1.4	2.94 ± 1.27	.683	−1.60581	1.07914
NPV (mm^3^)	4782.78 ± 4907.23	3642.78 ± 2203.75	.538	−2802.26106	5082.26106
OPV (cc)	5.74 ± 3.82	5.01 ± 4.91	0.7294	−3.665	5.131
Min area (cc)	149.92 ± 114.69	56.21 ± 63.22	0.0475*	−186.2	−1.173

Data presented as Mean ± SD

Used test: Unpaired t test

*Statistically significant at p ≤ 0.05

##### Pain

The comparison between the pain scale in the first two weeks and the second two weeks in both groups is shown in Table 3. Moderate pain was experienced in the first two weeks of expansion in MMG (5.11 ± 0.30), while severe pain was recorded in NMG (6.87 ± 0.40). Pain decreased significantly in the following two weeks in both groups with mild pain in MMG (2.77 ± 0.39) and moderate pain in NMG (5.11 ± 0.32) with significant difference between the two groups along the entire duration of expansion.

**Table 3:** The comparison between pain scale in the first two weeks and the second two weeks in both groups

Measurement	NMG		MMG		*P* value	95% CI
	1st two weeks	2nd two weeks	1st two weeks	2nd two weeks		
VAS	6.87 ± 0.40	5.11 ± 0.32	5.11 ± 0.30	2.77 ± 0.39	P1 < 0.0001* P2 < 0.0001*	−1.948 to −1.569 −2.525 to −2.140
*P* value	P < 0.0001*		*P* < 0.0001*			
95% CI	−1.971 to −1.548		−2.567 to −2.100			

Data presented as Mean ± SD

P: Statistical significance between the two time points, P1: Statistical significance between the two groups during the 1st two weeks, P2: Statistical significance between the two groups during the 2nd two weeks

Used test: Unpaired t test to compare between the two studied groups, and Paired t test to compare between the two time points in the same group

^*^Statistically significant at *p* ≤ 0.05

### Reliability testing

The intra-observer absolute agreement was measured using the intraclass correlation coefficient (ICC). A high degree of reliability was found with an average measure of 0.902.

### Harms

During the trial, no significant risks were observed, except discomfort experienced by some patients. Some negative effects were observed, like the palate's soft tissue compression, indentations in the tongue, and instability of the posterior mini-screws.

## Discussion

The study was registered in ClinicalTrials after its initiation. It ideally should be registered before its start. However, no work was done before the study protocol was registered at Mansoura University and was approved by the "Dental Research Ethics Committee" of the Mansoura University Faculty of Dentistry in August 2022. We tried to overcome this flaw by strictly following the guidelines without any violations and by reporting the trial with high transparency.

### Airway

This study assesses the impact of MARPE that was facilitated by MOP at the MPS on the airway volume. The study's sample revealed uniformity in the demographic information (age and sex). Additionally, none of the preoperative data showed a statistically significant difference between the two groups. In addition, the anterior and posterior opening of the suture after expansion did not differ significantly in the two groups. Since the intended amount of expansion was similar in both groups, the lack of changes at the commencement of the treatment and after expansion allowed for an acceptable comparison of the findings.

Maxillary expansion's impact on the nasal cavity and other pharyngeal airway regions has been shown in recent studies [19–21].Yet, there is disagreement over the regions and sub-regions assessed, as well as the assessment techniques [22, 23]. The technique employed in this investigation allowed for sufficient accuracy in terms of the anatomical locations where linear measurements were made.

Both techniques, with MOP or without it, were successful in treating transverse maxillary deficit, exhibiting comparable nasal skeletal effects. The NCW and nasopharyngeal volume were wider after MARPE than before treatment in both groups, which is advantageous for patients with upper airway ventilation, despite the fact that no breathing test was performed, and no control group was established due to ethical limits. There was a more but insignificant increase in all the measurements of the nasal cavity in NMG compared to MMG except for the minimum area which increased significantly more in NMG. So, MOP did not add any further advantage in improving the airway volume after maxillary expansion.

This treatment effect supported the results of an earlier investigation that found an 8.48% increase in nasopharyngeal volume [24]. This implies that the transverse expansion of bone caused by MARPE may have incidental consequences on the upper airway. This finding showed that the secondary response of MARPE was comparable to the outcomes of both surgically aided RME in adults and traditional RME in children [25, 26].

According to recent research [11–13], MARPE dramatically expands the young patients' minimum cross-sectional area and airway volume.

Regardless of maturity stage, Aneris et al. [27] observed a significant increase in all volumetric metrics, including minimal constriction area, following expansion with MARPE.

WAR, WMR, WPR, AOS, POS, and volumetric airway measurements were found to significantly increase in MISMARPE (minimally invasive surgically aided MARPE) & SARPE, according to Bastos et al. [28].

De Felippe et al. [29] saw a notable 30.12% increase in the volume of the nasal cavities, which reduced nasal resistance and enhanced ventilation following rapid maxillary extension.

On the contrary, Ribeiro et al. [30] found no change in the nasopharyngeal airway volume despite a 239 mm^3^increase in the oropharyngeal airway volume. The author speculates that this could be because the head and tongue were not in a standardized position during the image acquisition [31].

Also, Shetty et al. [32] noted a statistically insignificant decrease in both the overall airway length and volume following MARPE. This underscores the limitations of CBCT, particularly with regard to precisely measuring the volume of the anatomically and morphologically convoluted airway.

It's critical to note that in the current study, the choice was to do CBCT three months following the start of active treatment in order to prevent transient side effects like edema or clockwise mandibular rotation, which could produce inaccurate results [33].

### Pain perception

Physical pain, defined as the incidence of severe aching and discomfort during eating, was observed to considerably increase after expansion with MARPE [34].

Given their regularity and approval as a form of intervention, tooth extractions are frequently used as a reference operation for the measurement of pain intensity from the perspective of orthodontic management [35]. Following premolar extraction, Ganzer et al. [36] observed a mean VAS score of 38.50 mm, whereas Chen et al. [35] measured a score of 35.80 mm. By contrast, during the first two weeks of expansion, the average pain from the MARPE treatment was 5.11 mm for MMG and 6.87 mm for NMG, which is comparable to the discomfort after premolar extractions and can be deemed minimally hurting. Kapetanovic et al. [34] recorded a mean VAS score of 33.72 mm for typical pain during the first week of MARPE treatment. Additionally, 24 h following the placement of fixed orthodontic appliances, Tseng et al. [37] observed a pain VAS-score of 36.3 mm, which is greater than the pain MARPE induced at the beginning. Pain decreased significantly in the second two weeks of expansion with significantly lower mean values in the MOP group (2.77 mm) than that found when MOP was not performed (5.11 mm).

Regarding the intake of analgesics, only two patients reported unbearable pain and took analgesics; thus, they were considered dropouts and were not included in the analysis.

In terms of how pain evolved throughout MARPE, it was comparable to other non-surgical RPE therapies, which produced discomfort in 98% of patients. The most pain was experienced during the first week of expansion when it was at its highest, and then it steadily reduced after that [38, 39].

### Limitations

Patient perception measurements were intrinsically subjective and dependent on the ability to convert a subjective experience into an objective measure that was affected by interindividual variances. The mean VAS scores were compared with traditional orthodontic and surgical (expansion) procedures to enable a clinically appropriate interpretation. Moreover, there might have been no control over the tongue's position during the CBCT scans, which is a crucial anatomical component influencing the dimensions and form of the oropharyngeal airway volume.

### Generalizability

The study's generalisability may be limited by the fact that the treatments were administered to a single ethnic group by a single dental institution and one PhD candidate.

## Conclusion

Both techniques of expansion, with or without MOP, were successful in treating transverse maxillary deficit, with comparable nasal skeletal effects. Both techniques greatly enhanced the volumetric characteristics in the upper airway spaces. So, MOP did not add any further advantage in improving the airway volume after maxillary expansion. However, with MOP, pain intensity was greatly reduced in the first two weeks of expansion, and it was further reduced in the following two weeks.

## Data Availability

All the datasets used and analyzed during the current study are available from the corresponding author on reasonable request.

## Abbreviations

MARPE: Mini-screw assisted rapid palatal expansion
MOP: Micro-osteoperforation
MMG: MARPE with MOP group
NMG: MARPE with no MOP group
VAS: Visual Analogue Scale
RCT: Randomized clinical trial
RPE: Rapid palatal expansion
MPS: Mid-palatal suture
CBCT: Cone-beam computed tomography
MSE II: Maxillary Skeletal Expander®

## References

[1] Lim H-M, Park Y-C, Lee K-J, Kim K-H, Choi YJ. Stability of dental, alveolar, and skeletal changes after miniscrew-assisted rapid palatal expansion. Korean J Orthod. 2017;47(5):313–22. 28861393 10.4041/kjod.2017.47.5.313 PMC5548712

[2] Lee K-J, Park Y-C, Park J-Y, Hwang W-S. Miniscrew-assisted nonsurgical palatal expansion before orthognathic surgery for a patient with severe mandibular prognathism. Am J Orthod Dentofac Orthop. 2010;137(6):830–9. 10.1016/j.ajodo.2007.10.065 20685540

[3] Carlson C, Sung J, McComb RW, Machado AW, Moon W. Microimplant-assisted rapid palatal expansion appliance to orthopedically correct transverse maxillary deficiency in an adult. Am J Orthod Dentofac Orthop. 2016;149(5):716–28. 10.1016/j.ajodo.2015.04.043 27131254

[4] Choi S-H, Shi K-K, Cha J-Y, Park Y-C, Lee K-J. Nonsurgical miniscrew-assisted rapid maxillary expansion results in acceptable stability in young adults. Angle Orthod. 2016;86(5):713–20. 26938955 10.2319/101415-689.1 PMC8600851

[5] Persson M, Thilander B. Palatal suture closure in man from 15 to 35 years of age. Am J of Orthod. 1977;72(1):42–52. 267435 10.1016/0002-9416(77)90123-3

[6] Suri L, Taneja P. Surgically assisted rapid palatal expansion: a literature review. Am J Orthod Dentofac Orthop. 2008;133(2):290–302. 10.1016/j.ajodo.2007.01.021 18249297

[7] Echchadi ME, Benchikh B, Bellamine M, Kim S-H. Corticotomy-assisted rapid maxillary expansion: A novel approach with a 3-year follow-up. Am J Orthod Dentofac Orthop. 2015;148(1):138–53. 10.1016/j.ajodo.2014.08.023 26124037

[8] Hassan AH, AlGhamdi AT, Al-Fraidi AA, Al-Hubail A, Hajrassy MK. Unilateral cross bite treated by corticotomy-assisted expansion: two case reports. Head Face Med. 2010;6:1–9. 20482859 10.1186/1746-160X-6-6 PMC2893126

[9] Hassan AH, Al-Fraidi AA, Al-Saeed SH. Corticotomy-assisted orthodontic treatment. Open. Dent J. 2010;4:159. 10.2174/1874210601004010159 PMC3019587 21228919

[10] Suzuki SS, Braga LFS, Fujii DN, Moon W, Suzuki H. Corticopuncture Facilitated Microimplant-Assisted Rapid Palatal Expansion. Case rep dent. 2018;2018(1):1392895. 30627452 10.1155/2018/1392895 PMC6305058

[11] Kim S-Y, Park Y-C, Lee K-J, Lintermann A, Han S-S, Yu H-S, et al. Assessment of changes in the nasal airway after nonsurgical miniscrew-assisted rapid maxillary expansion in young adults. Angle Orthod. 2018;88(4):435–41. 29561652 10.2319/092917-656.1 PMC8191933

[12] Mehta S, Wang D, Kuo C-L, Mu J, Vich ML, Allareddy V, et al. Long-term effects of mini-screw–assisted rapid palatal expansion on airway: A three-dimensional cone-beam computed tomography study. Angle Orthod. 2021;91(2):195–205. 33315060 10.2319/062520-586.1 PMC8028479

[13] Capelozza Filho L, Da Silva FO. Rapid maxillary expansion: a general approach and clinical applications. Part I Dental Press J Orthod. 1997;2(3):88–102.

[14] Kapetanović A, Theodorou CI, Bergé SJ, Schols JG, Xi T. Efficacy of Miniscrew-Assisted Rapid Palatal Expansion (MARPE) in late adolescents and adults: a systematic review and meta-analysis. Eur J Orthod. 2021;43(3):313–23. 33882127 10.1093/ejo/cjab005 PMC8186837

[15] Brunetto DP, Sant’Anna EF, Machado AW, Moon W. Non-surgical treatment of transverse deficiency in adults using Microimplant-assisted Rapid Palatal Expansion (MARPE). Dental Press J Orthod. 2017;22:110–25. 28444019 10.1590/2177-6709.22.1.110-125.sar PMC5398849

[16] Sangsuwon C, Alansari S, Lee Yb, Nervina J, Alikhani M. Step-by-step guide for performing micro-osteoperforations. Clinical Guide to Accelerated Orthodontics: With a Focus on Micro-Osteoperforations. 2017:99–116.

[17] Emshoff R, Bertram S, Emshoff I. Clinically important difference thresholds of the visual analog scale: a conceptual model for identifying meaningful intraindividual changes for pain intensity. PAIN®. 2011;152(10):2277–82. 21726939 10.1016/j.pain.2011.06.003

[18] de Oliveira CB, Ayub P, Ledra IM, Murata WH, Suzuki SS, Ravelli DB, et al. Microimplant assisted rapid palatal expansion vs surgically assisted rapid palatal expansion for maxillary transverse discrepancy treatment. Am J Orthod Dentofac Orthop. 2021;159(6):733–42. 10.1016/j.ajodo.2020.03.024 33931257

[19] da Silva AV, da Rosa BM, Matje PR, Rizzatto SMD, de Oliveira RB, Haas OL Jr, et al. Effects of SARPE and MISMARPE on correction of transverse maxillary deficiency: A preliminary comparative evaluation. J Orthod Craniofac Res. 2024;27(2):332–8. 10.1111/ocr.12712 37728033

[20] Heldmaier W, Lonic D, Loeffelbein DJ. Three-dimensional analyses of postoperative effects of surgically assisted rapid palatal expansion (SARPE) on the soft tissue of the midface region and the upper airway space using stereophotogrammetry and cone beam computed tomography (CBCT). Am Surg. 2023;89(4):553–7. 36622309 10.1177/00031348221148349

[21] Jia H, Zhuang L, Zhang N, Bian Y, Li S. Comparison of skeletal maxillary transverse deficiency treated by microimplant-assisted rapid palatal expansion and tooth-borne expansion during the post-pubertal growth spurt stage: A prospective cone beam computed tomography study. Angle Orthod. 2021;91(1):36–45. 33289835 10.2319/041920-332.1 PMC8032284

[22] Kober C, Kannenberg S, Frank B, Al-Hakim G, Parvin A, Landes C, et al. Computer-assisted pre-and postoperative evaluation of surgically assisted rapid maxillary expansion. Int J Comput Dent. 2011;14(3):233–41. 22141233

[23] Andriola FdO, Haas Junior OL, Guijarro-Martinez R, Hernandez-Alfaro F, Oliveira RBd, Pagnoncelli RM, et al. Computed tomography imaging superimposition protocols to assess outcomes in orthognathic surgery: a systematic review with comprehensive recommendations. Dentomaxillofac Radiol. 2022;51(3):20210340. 34520241 10.1259/dmfr.20210340 PMC8925870

[24] Yi F, Liu S, Lei L, Liu O, Zhang L, Peng Q, et al. Changes of the upper airway and bone in microimplant-assisted rapid palatal expansion: A cone-beam computed tomography (CBCT) study. J Xray Sci Technol. 2020;28(2):271–83. 31985485 10.3233/XST-190597

[25] Suzuki H, Moon W, Previdente LH, Suzuki SS, Garcez AS, Consolaro A. Miniscrew-assisted rapid palatal expander (MARPE): the quest for pure orthopedic movement. Dental Press J Orthod. 2016;21:17–23. 27653260 10.1590/2177-6709.21.4.017-023.oin PMC5029312

[26] Fastuca R, Perinetti G, Zecca PA, Nucera R, Caprioglio A. Airway compartments volume and oxygen saturation changes after rapid maxillary expansion: a longitudinal correlation study. Angle Orthod. 2015;85(6):955–61. 26516709 10.2319/072014-504.1 PMC8612044

[27] Anéris FF, El Haje O, Rosário HD, de Menezes CC, Franzini CM, Custodio W. The effects of miniscrew-assisted rapid palatal expansion on the upper airway of adults with midpalatal suture in the last two degrees of ossification. J World Fed Orthod. 2023;12(4):150–5. 37344294 10.1016/j.ejwf.2023.05.005

[28] Bastos R, Junior OH, Piccoli V, da Rosa B, de Oliveira R, de Menezes L. Effects of minimally invasive surgical and miniscrew-assisted rapid palatal expansion (MISMARPE) on the nasal cavity and upper airway: a comparative cohort study. Int J Oral Maxillofac Surg. 2024;xx:1–8. 10.1016/j.ijom.2024.03.012 38609790

[29] De Felippe NLO, Da Silveira AC, Viana G, Kusnoto B, Smith B, Evans CA. Relationship between rapid maxillary expansion and nasal cavity size and airway resistance: short-and long-term effects. Am J Orthod Dentofac Orthop. 2008;134(3):370–82. 10.1016/j.ajodo.2006.10.034 18774083

[30] Ribeiro ANC, De Paiva JB, Rino-Neto J, Illipronti-Filho E, Trivino T, Fantini SM. Upper airway expansion after rapid maxillary expansion evaluated with cone beam computed tomography. Angle Orthod. 2012;82(3):458–63. 21999215 10.2319/030411-157.1 PMC8865811

[31] Aziz T, Ansari K, Lagravere MO, Major MP, Flores-Mir C. Effect of non-surgical maxillary expansion on the nasal septum deviation: a systematic review. Prog Orthod. 2015;16:1–7. 26061988 10.1186/s40510-015-0084-y PMC4456578

[32] Shetty A, Ratti S, Nakra P, Shetty S, Mohammed A, Saidath K. Evaluation of soft tissue and airway changes in individuals treated with Mini-Implant Assisted Rapid Palatal Expansion (MARPE). J Long Term Eff Med Implants. 2022;32(1):7–18. 10.1615/JLongTermEffMedImplants.2021038874 35377989

[33] Chang Y, Koenig LJ, Pruszynski JE, Bradley TG, Bosio JA, Liu D. Dimensional changes of upper airway after rapid maxillary expansion: a prospective cone-beam computed tomography study. Am J Orthod Dentofac Orthop. 2013;143(4):462–70. 10.1016/j.ajodo.2012.11.019 23561406

[34] Kapetanović A, Noverraz RR, Listl S, Bergé SJ, Xi T, Schols JG. What is the oral health-related quality of life following miniscrew-assisted rapid palatal expansion (MARPE)? A prospective clinical cohort study. BMC Oral Health. 2022;22(1):423. 36138473 10.1186/s12903-022-02444-3 PMC9502924

[35] Chen C-M, Chang C-S, Tseng Y-C, Hsu K-R, Lee K-T, Lee H-E. The perception of pain following interdental microimplant treatment for skeletal anchorage: a retrospective study. Odontology. 2011;99:88–91. 21271332 10.1007/s10266-010-0152-1

[36] Ganzer N, Feldmann I, Bondemark L. Pain and discomfort following insertion of miniscrews and premolar extractions: a randomized controlled trial. Angle Orthod. 2016;86(6):891–9. 27023407 10.2319/123115-899.1 PMC8597328

[37] Tseng Y-C, Chen C-M, Wang H-C, Wang C-H, Lee H-E, Lee K-T. Pain perception during miniplate-assisted orthodontic therapy. Kaohsiung J Med Sci. 2010;26(11):603–8. 21126713 10.1016/S1607-551X(10)70092-9 PMC11916556

[38] Pithon MM, Marañón-Vásquez GA, da Silva LP, da Silva CR, Dos Santos RL, Tanaka OM, et al. Effect of treatment of transverse maxillary deficiency using rapid palatal expansion on oral health-related quality of life in children: A randomized controlled trial. Am J Orthod Dentofac Orthop. 2022;161(2):172–81. 10.1016/j.ajodo.2021.08.015 34711482

[39] Serritella E, Migliaccio S, Musone L, Impellizzeri A, De Stefano AA, Galluccio G. Perceived pain during rapid maxillary expansion (RME): trends, anatomical distinctions, and age and gender correlations. Pain Res Manag. 2021;2021(1):7396466. 34336069 10.1155/2021/7396466 PMC8295004

